# Potential of *Cladophialophora inabaensis* EUCL1 and *Exophiala* sp. BCM1 for abiotic stress resilience in maize

**DOI:** 10.7717/peerj.19947

**Published:** 2026-01-28

**Authors:** Ni Luh Putu Citra Innosensia, Haoyue Lu, Kazuhiko Narisawa

**Affiliations:** Department of Bioresource Science, College of Agriculture, Ibaraki University, Ami, Ibaraki, Japan

**Keywords:** Drought, Saline, Alkaline salt, Abiotic stress, Dark septate endophytic fungi, Plant-microbe interaction

## Abstract

This study investigated the effects of two dark septate endophytic (DSE) fungi, *Cladophialophora inabaensis* EUCL1 and *Exophiala* sp. BCM1, on maize growth under no-stress, drought, saline, and alkaline salt conditions. Maize was cultivated in agar and soil-based systems, and growth parameters including shoot and root lengths, biomass, chlorophyll content, and stem diameter were evaluated to assess the efficacy of DSE inoculation. Both *C. inabaensis* EUCL1 and *Exophiala* sp. BCM1 showed promising effects to ameliorate negative effects of drought, saline, and alkaline salt stress. Maize inoculated with *C. inabaensis* EUCL1 exhibited significantly enhanced growth under no-stress conditions. Under drought stress, *C. inabaensis* EUCL1 increased shoot length by 148.94% *in vitro*, while *Exophiala* sp. BCM1 improved shoot and root dry mass by 196.55 and 188.21% respectively, on soil cultivation compared with the control. Notably, *C. inabaensis* EUCL1 also demonstrated strong potential in supporting maize growth under both saline and alkaline salt stress in soil-based systems. In response to saline stress, *C. inabaensis* EUCL1-treated plants exhibited marked increases in shoot and root dry mass by 176.15 and 152.77%, respectively. Under alkaline salt stress, shoot and root dry mass increased by 352.28 and 153.3%, respectively, compared with the control. Overall improvements in observed growth parameters indicate that DSE inoculation successfully mitigated the negative effects of abiotic stress. This study is the first to report the efficacy of *C. inabaensis* EUCL1 and *Exophiala* sp. BCM1 as effective bioinoculants for enhancing maize resilience under multiple abiotic stresses.

## Introduction

Climate change-induced stressors severely threaten the growth and productivity of maize (*Zea mays* L.), jeopardizing global food security (Boyer et al., 2013; Zampieri et al., 2019; Salika & Riffat, 2021; Klein & Luna, 2022). Climate anomalies intensify drought, salinity, and alkalinity, which are often interrelated in arid and semi-arid regions, where water scarcity promotes salt accumulation and subsequent soil alkalinization, compounding their adverse effects on plant performance (Cominelli et al., 2013; Parihar et al., 2015; Zhang et al., 2022). Drought stress adversely affects maize seedling development through downregulation of photosynthesis and nutrient metabolism pathways, while also disrupting root architecture and reducing leaf formation during reproductive stages (Zhang et al., 2018; Sah et al., 2020). As a moderately salt-sensitive crop, maize shows significant reductions in plant height and total biomass under salt stress, along with decreased photosynthetic activity and increased oxidative damage (Munns, 2005; Farooq et al., 2015; Ouhaddou et al., 2023; Vennam et al., 2024). Additionally, increasing salt concentrations raise soil pH, leading to alkalization. Alkaline salt stress markedly suppress levels of photosynthesis, N metabolism, glycolysis, and production of sugars and amino acids in maize (Guo et al., 2017).

To address these climate-related challenges, it is essential to enhance maize performance through sustainable and environmentally friendly approaches that minimize further ecological damage. Emerging studies highlight beneficial microbes to support plant growth under harsh conditions, with dark septate endophytic (DSE) fungi being among the most promising candidates. This group of fungi is known to colonize while promoting plant growth without causing adverse effects on their host plants. They belong to ascomycetes and are characterized by melanized, septate hyphae and a microsclerotia structure (Jumpponen & Trappe, 1998). Their hyphae colonize plant roots both intra- and intercellularly, possibly resulting in improved plant growth, accelerated seed germination, induced systemic resistance, enhanced tolerance to abiotic stressors, and increased uptake of mineral nutrients (Andrade-Linares & Franken, 2013).

Dark septate endophytic fungi reportedly play a critical role in improving plant tolerance to drought stress, generally by enhancing root morphology and development (Liu & Wei, 2019; Li et al., 2022). Despite the urgency and increasing trend of drought conditions worldwide, studies investigating DSE effects on maize under drought are scarce and limited to only those under mild conditions (Li et al., 2019). Previous studies reported the ability of DSE fungi to enhance plant growth under saline stress. However, their application to maize, a crop known to be sensitive to salinity, has yet to be specifically investigated. While the endophytic fungi *Stemphylium lycopersici* and *Aspergillus terreus* have been reported to ameliorate saline stress in maize (Ali et al., 2022; Siddiqui et al., 2022) the potential role of DSE fungi in this context remains largely unexamined. Furthermore, most existing studies have concentrated on neutral salt, and there is limited information on plant growth under alkaline salt stress, particularly in association with DSE fungi.

Over the last few decades scientists have been exploring sustainable solutions to mitigate climate-change related consequences in maize. However, no comprehensive study assessed the effectiveness of DSE isolates used in this research; thus, their beneficial roles in promoting maize growth under drought, saline, and alkaline salt stresses have gone unreported. Therefore, this study investigates the potential effects of *Cladophialophora inabaensis* EUCL1 and *Exophiala* sp. BCM1 on maize growth under abiotic stress conditions. Two distinct cultivation systems were employed: agar and soil-based, representing controlled and semi-natural environments, respectively. The findings reveal previously unreported traits of *C. inabaensis* EUCL1 and *Exophiala* sp. BCM1 in promoting maize growth under various stress conditions. This work contributes important new knowledge to our understanding of the beneficial effects of DSE fungi on maize and provides valuable insights into their potential application in sustainable agriculture, particularly in addressing climate change-related challenges.

## Materials & Methods

### Fungal isolates

*Cladophialophora inabaensis* EUCL1 and *Exophiala* sp. BCM1 were obtained from the culture collection of microbial ecology laboratory, Ibaraki University, Japan. *Cladophialophora inabaensis* EUCL1 was isolated from a secondary forest soil in Tottori, Japan (Usui, Takashima & Narisawa, 2016). The isolate has been preserved in the NARO Genebank (MAFF 245257) in a metabolically inactive state. *Exophiala* sp. BCM1 has not been deposited in any public platform and remains part of the collection of the microbial ecology laboratory, where it was identified prior to this study.

### Screening of drought, saline, and alkaline salt stress concentrations for maize

The maize variety used in this study was Gold Rush 90 from Sakata Seed Corporation, Japan. Polyethylene glycol (PEG), sodium chloride (NaCl), and sodium carbonate (Na_2_CO_3_) were used to simulate drought, saline, and alkaline salt conditions, respectively. This experiment aimed to determine concentrations that inhibit maize growth while maintaining sufficient viability for subsequent analysis. Seeds were surface sterilized by soaking in sterile distilled water (SDW) for 12 h. They were then immersed in 75% alcohol for 1.5 min, 2% sodium hypochlorite for 1 min, and washed three times with SDW for 1 min each. Sterilized seeds were dried and then transferred onto 0.5% water agar for germination and subsequently transferred onto basic oatmeal medium (10 g oatmeal, 15 g Bacto agar, 1 g MgSO_4_⋅ 7H_2_O, 1.5 g KH_2_PO_4_, 1 g NaNO_3_, and 1 L distilled water) adjusted with different concentrations of PEG, NaCl, and Na_2_CO_3_.

For drought stress, PEG was added to the basic oatmeal medium at concentrations of 5, 10, 15, and 20%. As PEG does not solidify with agar, gellan gum was used instead of Bacto agar. For saline stress, NaCl was added at concentrations of 50, 100, 150, 250, and 300 mM. For alkaline salt stress, Na_2_CO_3_ was added at concentrations of 2 (pH 5.91), 4 (pH 6.47), 8 (pH 7.73), 16 (pH 8.34), and 32 mM (pH 9.15). Maize grown on basic oatmeal medium only was used as a control. Maize was grown under these conditions with 6 biological replications for each concentration gradient. The plants were grown axenically at 23 °C under a 16/8 (light/dark) cycle. At 20 days after sowing, the shoot and root lengths, and dry masses were measured.

### Effects of *Cladophialophora inabaensis* EUCL1 and *Exophiala* sp. BCM1 on maize under no-stress, drought, saline, and alkaline salt conditions: *in vitro* assay

Maize seeds were sterilized and germinated following the same procedures previously described in ‘Screening of drought, saline, and alkaline salt stress concentrations for maize’. Oatmeal medium supplemented with stress-inducing substances was prepared (drought: 10% PEG, saline: 100 mM NaCl, and alkaline: 8 mM Na_2_CO_3_). This was followed by the inoculation of medium with *C. inabaensis* EUCL1 and *Exophiala* sp. BCM1 for DSE treatments, while uninoculated medium was prepared for the non-treated control (NTC). Both isolates were incubated for two weeks to allow mycelial growth. Once the medium was covered with DSE, two germinated seeds were transplanted onto the medium and placed inside a sterile pot. Each treatment within each environment consisted of three pots, and the experiment was repeated three times using a completely randomized design. Incubation conditions followed those described in ‘Screening of drought, saline, and alkaline salt stress concentrations for maize’. After 20 days, a total of 18 biological replicates per treatment across all tested environments were harvested to assess plant length and biomass.

### Effects of *Cladophialophora inabaensis* EUCL1 and *Exophiala* sp. BCM1 on maize under no-stress, drought, saline, and alkaline salt conditions: soil system assay

#### Soil, DSE materials, and seedling preparation

Soil used in this study was a mixture of organic field soil and commercial river sand in a 3:1 ratio (v/v) (Lin et al., 2018). *Cladophialophora inabaensis* EUCL1 and *Exophiala* sp. BCM1 materials were prepared following a previously described method (Innosensia et al., 2023). Surface-sterilized maize seeds were sown in seedling trays with one of three treatments: (1) NTC: non-treated control soil, (2) EUCL1: soil mix with *C. inabaensis* EUCL1, and (3) BCM1: soil mix with *Exophiala* sp. BCM1. Soil and DSE materials were mixed at a ratio of 9:1. Maize was incubated in a growth chamber under a 16/8 (light/dark) cycle at a temperature of 23 °C for 15 days. Seedling germination was monitored daily and recorded until day 15. To examine the frequency of DSE colonization, a re-isolation method was used (Innosensia et al., 2023). Seedlings were then transferred into plastic pots (one kg of soil per pot) and placed in a greenhouse. Transplanted seedlings were allowed to adapt without any stress treatment for 5 days, with soil moisture content maintained at 25%.

#### Drought, saline, and alkaline salt stress conditions

For drought stress, the soil moisture content of all plants was reduced to 12%, which was maintained using electronic scales to weigh and adjust the water daily (Topp, Parkin & Ferre, 2007). To induce saline stress, salt in the form of NaCl solution was poured into the soil. Plants were watered with 100 mL of 100 mM NaCl solution every 2 days, resulting in a total addition of 8.77 g/kg NaCl. For alkaline salt stress, salt in the form of Na_2_CO_3_ solution was poured into the soil. Plants were watered with 100 mL of 8 mM Na_2_CO_3_ solution every 2 days, resulting in a total addition of 1.27 g/kg Na_2_CO_3_ (pH 7.50). Each treatment within an environment consisted of four pots, and the experiment was repeated three times using a randomized complete block design. After 30 days, a total of 12 biological replicates per treatment were assessed for shoot length (cm), root length (cm), shoot dry mass (mg), root dry mass (mg), chlorophyll content (SPAD), and stem diameter (mm).

### Statistical analysis

Stress concentration screening was analyzed using one-way ANOVA, followed by Tukey’s HSD test at a significance level of 0.05. For subsequent *in vitro* and soil-based experiments, the non-parametric Kruskal-Wallis test was applied, followed by Dunn’s test for pairwise comparisons at a significance level of 0.05. All statistical analyses and graphical visualizations were performed using OriginPro, Version 2023 (OriginLab Corporation, Northampton, MA, USA).

## Results

### Screening of drought, saline, and alkaline salt stress concentrations for maize

**Figure 1:**
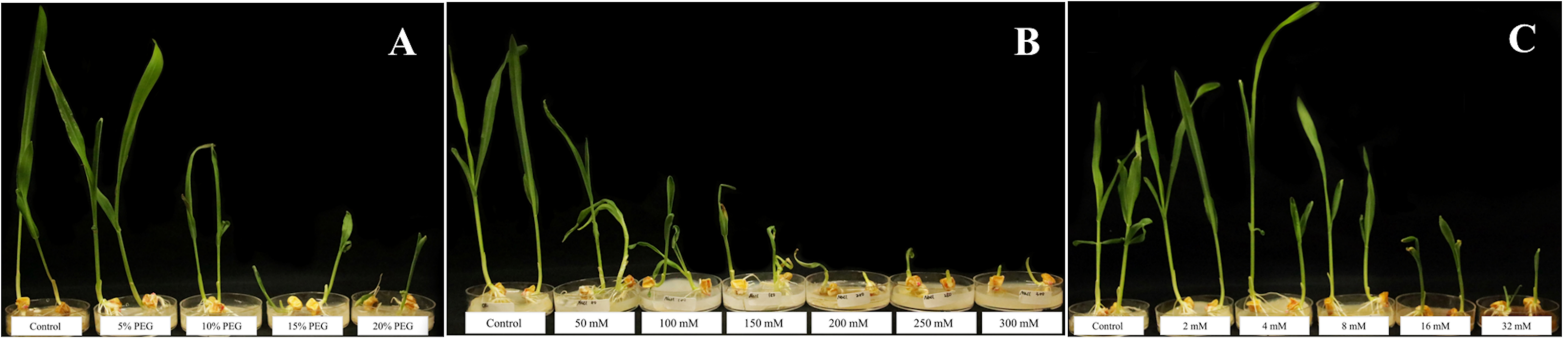
Abiotic stress concentration screening, showing maize responses under different stress levels. (A) Drought stress induced by PEG; (B) saline stress induced by NaCl; (C) alkaline salt stress induced by Na_2_CO_3_.

Table 1 shows marked reductions in maize shoot length and dry mass at 10% PEG compared with the control, with further declines at 15 and 20%. Specifically, 10% PEG caused a reduction in shoot length of 29.63% and decrease in shoot dry mass of 30.18% compared with the control. Figure 1A further shows that PEG concentrations from 10 to 20% resulted in reduced shoot performance compared with the control and 5% PEG. This indicates that 10% PEG is suitable for further drought stress examination, as it induced significant stress while still allowing sufficient plant survival for experimentation.

**Table 1:** The effects of drought stress on maize, under different PEG concentrations. The concentration in bold was used for subsequent experiments. Means followed by the same letter are not significantly different at *p* < 0.05 according to Tukey’s honestly significant difference test. Data are presented as mean ± standard error (SE).

PEG concentration /Parameter observation	Shoot length (cm)	Root length (cm)	Shoot dry mass (mg)	Root dry mass (mg)
Control	15.92 ± 1.28 a	13.17 ± 0.63 ab	50.92 ± 11.70 b	17.02 ± 1.56 b
5%	18.48 ± 0.66 a	15.03 ± 1.22 a	102.9 ± 9.63 a	34.57 ± 4.74 a
**10%**	**11.20 ± 0.58 b**	**15.18 ± 0.94 a**	**35.55 ± 4.96 bc**	**29.72 ± 3.49 ab**
15%	5.91 ± 0.52 c	11.55 ± 0.58 b	20.22 ± 2.88 c	26.73 ± 2.66 ab
20%	5.12 ± 0.42 c	10.28 ± 0.54 b	18.57 ± 2.58 c	20.37 ± 2.05 b

Table 2 shows saline concentration screening, indicating a significant reduction in shoot and root lengths beginning at 50 mM NaCl. At 100 mM, shoot length, root length, and shoot dry mass decreased significantly by 62.54, 51.83, and 57.85% respectively, compared with the control. From 150 mM onward, all growth parameters declined sharply, markedly impairing maize growth. The concentration of 100 mM NaCl was selected for subsequent experiments as it sufficiently inhibited maize growth without causing severe morphological damage. As shown in Fig. 1B, maize grown under 100 mM NaCl exhibited distinct morphological changes compared with the control.

**Table 2:** The effects of saline stress on maize, under different NaCl concentrations. The concentration in bold was used for subsequent experiments. Means followed by the same letter are not significantly different at *p* < 0.05 according to Tukey’s honestly significant difference test. Data are presented as mean ± standard error (SE).

NaCl concentration /Parameter observation	Shoot length (cm)	Root length (cm)	Shoot dry mass (mg)	Root dry mass (mg)
Control	17.48 ± 0.33 a	15.05 ± 0.71 a	51.92 ± 9.06 a	30.10 ± 2.67 a
50	13.27 ± 0.35 b	8.23 ± 0.56 b	41.98 ± 3.84 a	24.35 ± 1.57 ab
**100**	**6.55 ± 0.22 c**	**7.25 ± 0.54 b**	**21.88 ± 2.25 b**	**23.52 ± 1.53 ab**
150	6.20 ± 0.41 c	3.52 ± 0.22 c	21.48 ± 2.84 b	21.4 ± 2.11 b
200	3.13 ± 0.60 d	1.4 ± 0.17 d	11.33 ± 2.25 b	5.97 ± 0.81 c
250	2.20 ± 0.37 d	1.3 ± 0.23 d	12.08 ± 1.56 b	4.72 ± 0.96 c
300	1.52 ± 0.20 d	1.15 ± 0.14 d	6.03 ± 0.61 b	3.97 ± 0.40 c

At two and four mM Na_2_CO_3_, there were no significant differences in maize length or biomass compared with the control (Table 3). At eight mM Na_2_CO_3_, all observed parameters decreased compared with the control, with root length and shoot dry mass being significantly reduced. At this concentration, shoot length, root length, shoot dry mass, and root dry mass decreased by 11.40, 71.59, 62, and 19.39%, respectively, compared with the control. At 16 and 32 mM Na_2_CO_3_, maize performance sharply declined, severely impacting plants both above and below ground (Fig. 1C). Finally, 8 mM Na_2_CO_3_ was selected for further examination of alkaline salt stress, as it effectively induced stress without causing excessive damage.

**Table 3:** The effects of alkaline salt stress on maize, under different Na_2_CO_3_ concentrations. The concentration in bold was used for subsequent experiments. Means followed by the same letter are not significantly different at *p* < 0.05 according to Tukey’s honestly significant difference test. Data are presented as mean ± standard error (SE).

Na_2_CO_3_ concentration /Parameter observation	Shoot length (cm)	Root length (cm)	Shoot dry weight (mg)	Root dry weight (mg)
Control	14.18 ± 0.43 a	12.08 ± 0.40 a	80.18 ± 10.16 a	19.77 ± 2.69 abc
2	13.75 ± 0.58 a	11.88 ± 1.40 a	60.27 ± 7.47 a	22 ± 5.02 ab
4	14.03 ± 1.71 a	11.07 ± 1.90 a	58.33 ± 8.09 a	25.95 ± 4.22 a
**8**	**12.57 ± 1.01 a**	**3.43 ± 0.49 b**	**30.47 ± 3.08 b**	**15.93 ± 2.12 abc**
16	6.4 ± 0.57 b	1.5 ± 0.18 b	19.98 ± 1.38 b	9.65 ± 0.79 bc
32	2.95 ± 0.39 b	1.02 ± 0.08 b	8.92 ± 1.60 b	7.7 ± 0.40 c

### Effects of *Cladophialophora inabaensis* EUCL1 and *Exophiala* sp. BCM1 on maize under no-stress, drought, saline, and alkaline salt conditions: *in vitro* assay

**Figure 2:**
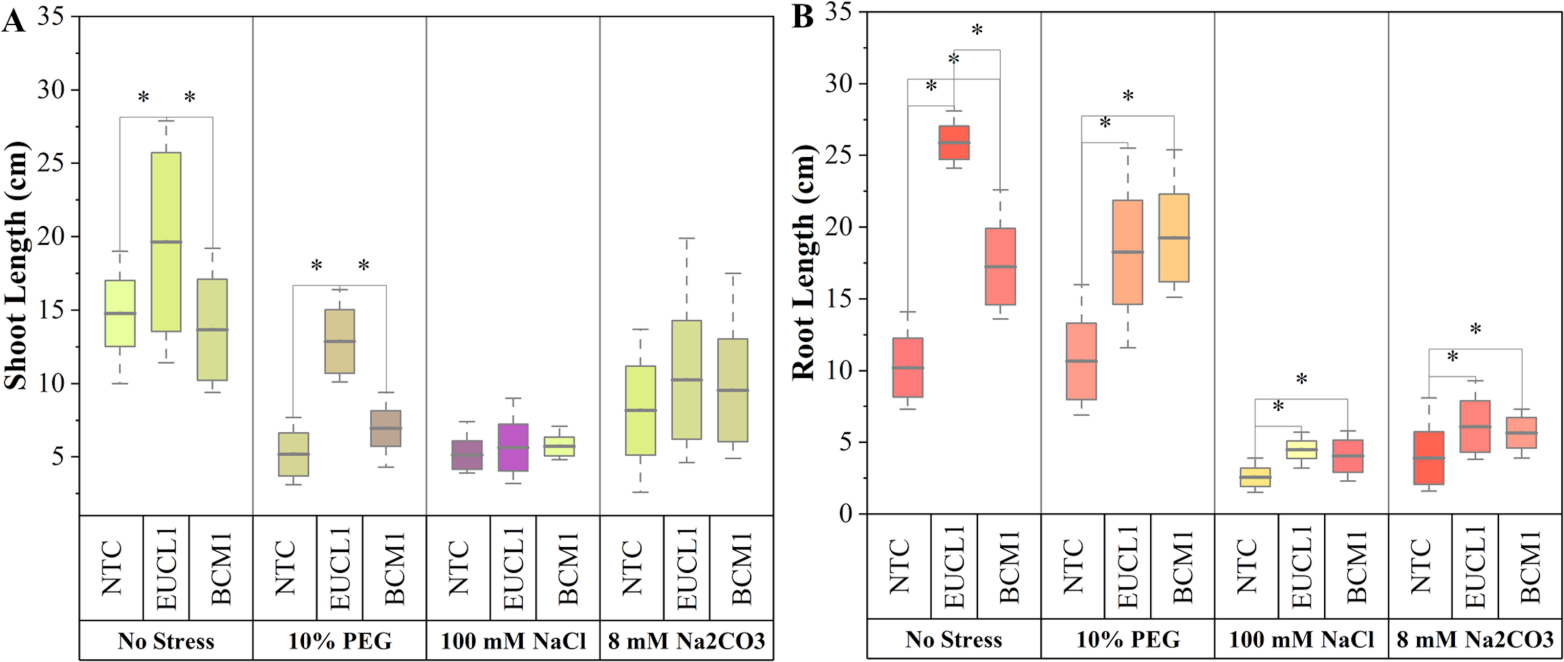
Enhanced maize lengths with *Cladophialophora inabaensis* EUCL1 and *Exophiala* sp. BCM1 treatments under all tested conditions. (A) Shoot length; (B) root length. Box plots represent the standard deviation (SD), with whiskers indicating minimum and maximum values. Horizontal lines within boxes show the mean. Asterisks (*) above connecting lines indicate significant differences between the two connected treatments at the 0.05 significance level.

Figure 2A illustrates maize shoot length under three different treatments across four independent environments. Under no-stress, *C. inabaensis* EUCL1 significantly increased maize shoot length by 43.60 and 32.90% compared with *Exophiala* sp. BCM1 and NTC, respectively. Under drought stress *C. inabaensis* EUCL1 enhanced maize shoot length by 85.45 and 148.94% compared with *Exophiala* sp. BCM1 and NTC, respectively. Under saline and alkaline salt stress, no significant differences were observed among treatments. Figure 2B shows the effects of DSE treatments on maize root length. Generally, all DSE treatments increased root length compared with NTC. *Cladophialophora inabaensis* EUCL1 significantly enhanced maize root length compared with NTC under no-stress, saline, and alkaline salt conditions by 153.73, 75, and 56.41%, respectively. Under drought stress, *Exophiala* sp. BCM1 achieved the greatest improvement, showing a significant 80.75% increase compared with NTC.

**Figure 3:**
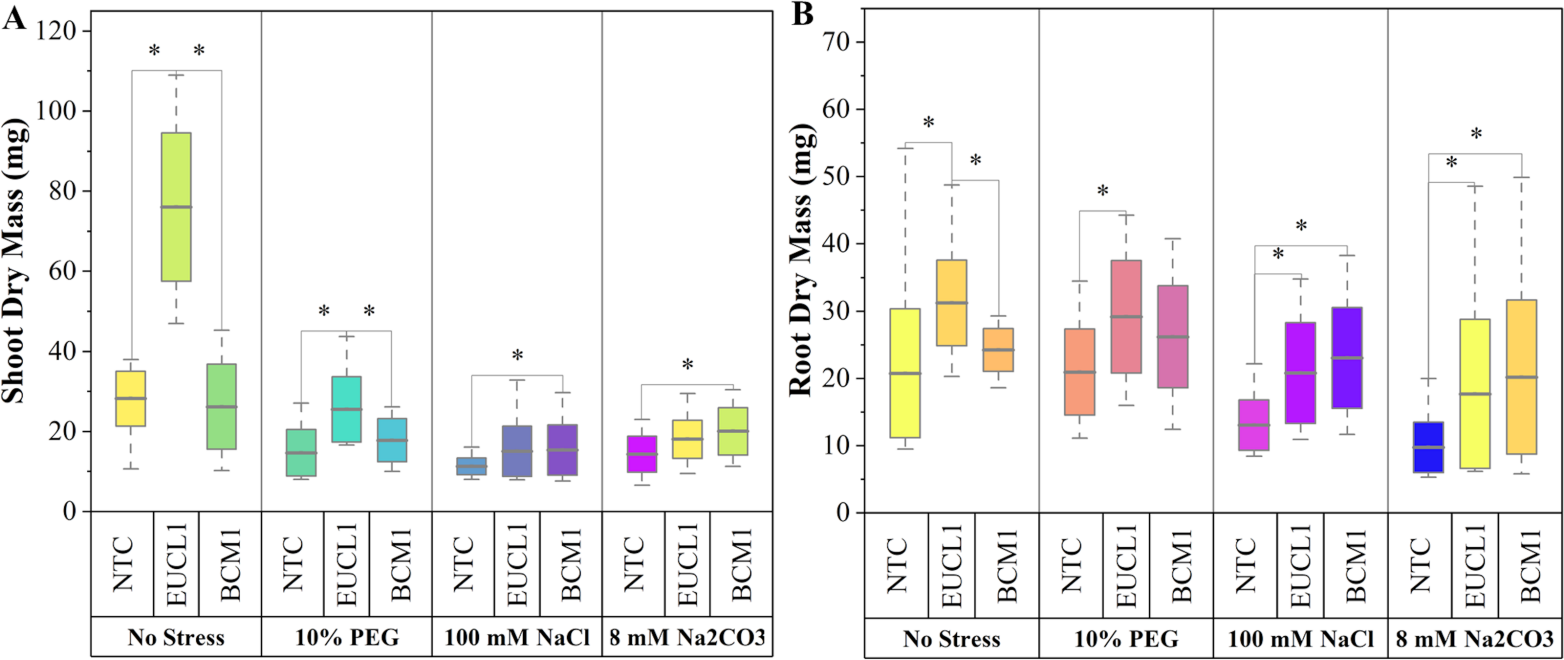
Enhanced maize dry masses with *Cladophialophora inabaensis* EUCL1 and *Exophiala* sp. BCM1 treatments under all tested conditions. (A) Shoot dry mass; (B) root dry mass. Box plots represent the standard deviation (SD), with whiskers indicating minimum and maximum values. Horizontal lines within boxes show the mean. Asterisks (*) above connecting lines indicate significant differences between the two connected treatments at the 0.05 significance level.

Figure 3A illustrates the effects of DSE treatments on maize shoot dry mass across various environmental conditions. Both DSE treatments generally increased shoot dry mass compared with NTC. *Cladophialophora inabaensis* EUCL1 significantly increased shoot dry mass under no-stress and drought conditions by 169.54 and 73.91%, respectively, compared with NTC. Under saline and alkaline salt stress, *Exophiala* sp. BCM1 led to significant increases of 36.22 and 39.37%, respectively, compared with NTC. Figure 3B demonstrates that DSE treatments consistently improved maize root dry mass relative to NTC. Under no-stress and drought, *C. inabaensis* EUCL1 significantly increased maize root dry mass compared with NTC by 50.41 and 39.30%, respectively. Under saline stress, both *Exophiala* sp. BCM1 and *C. inabaensis* EUCL1 significantly enhanced root dry mass compared with NTC by 76.53 and 59.59%, respectively. A similar trend was observed under alkaline salt stress, where *Exophiala* sp. BCM1 and *C. inabaensis* EUCL1 significantly increased root dry mass by 106.86 and 81.25%, respectively, compared with NTC.

### Effects of *Cladophialophora inabaensis* EUCL1 and *Exophiala* sp. BCM1 on maize under no-stress, drought, saline, and alkaline salt conditions: soil system assay

**Figure 4:**
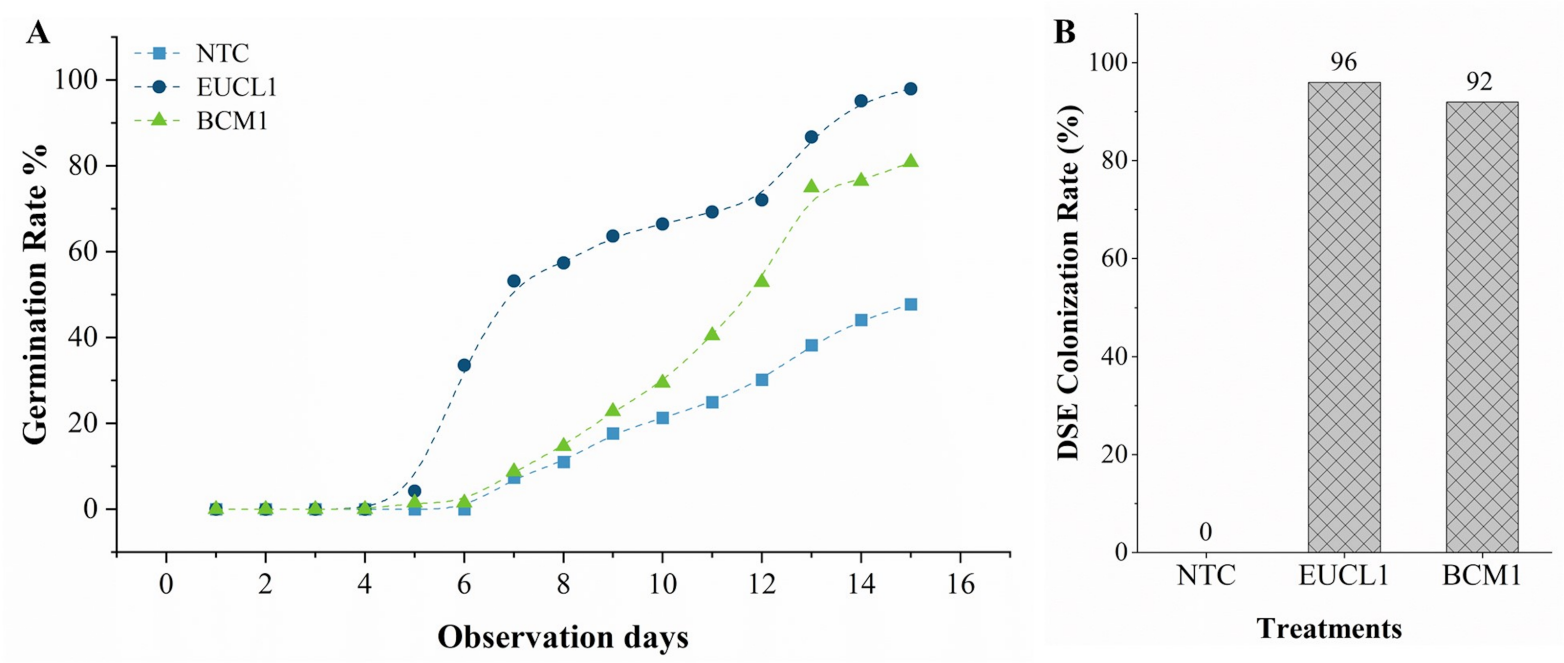
*Cladophialophora inabaensis* EUCL1 and *Exophiala* sp. BCM1 promote faster and greater germination, with the colonization rate reaching up to 96%. (A) Seedling germination rate; (B) DSE colonization rate (%).

Figure 4A illustrates the germination rate of maize grown in no-stress soil over 15 days. Maize treated with *C. inabaensis* EUCL1 germinated faster, reaching 50% by day 7, compared with *Exophiala* sp. BCM1 which achieved 50% germination by day 12. The NTC maize began germinating on day 6 and grew gradually. Final germination rates were: 47.79% for NTC, 80.88% for *Exophiala* sp. BCM1, and 97.90% for *C. inabaensis* EUCL1. Figure 4B shows DSE colonization rates in maize seedlings. NTC showed 0% colonization, indicating no cross-contamination during cultivation. Both *C. inabaensis* EUCL1 and *Exophiala* sp. BCM1 exhibited high colonization rates of 96 and 92%, respectively.

**Figure 5:**
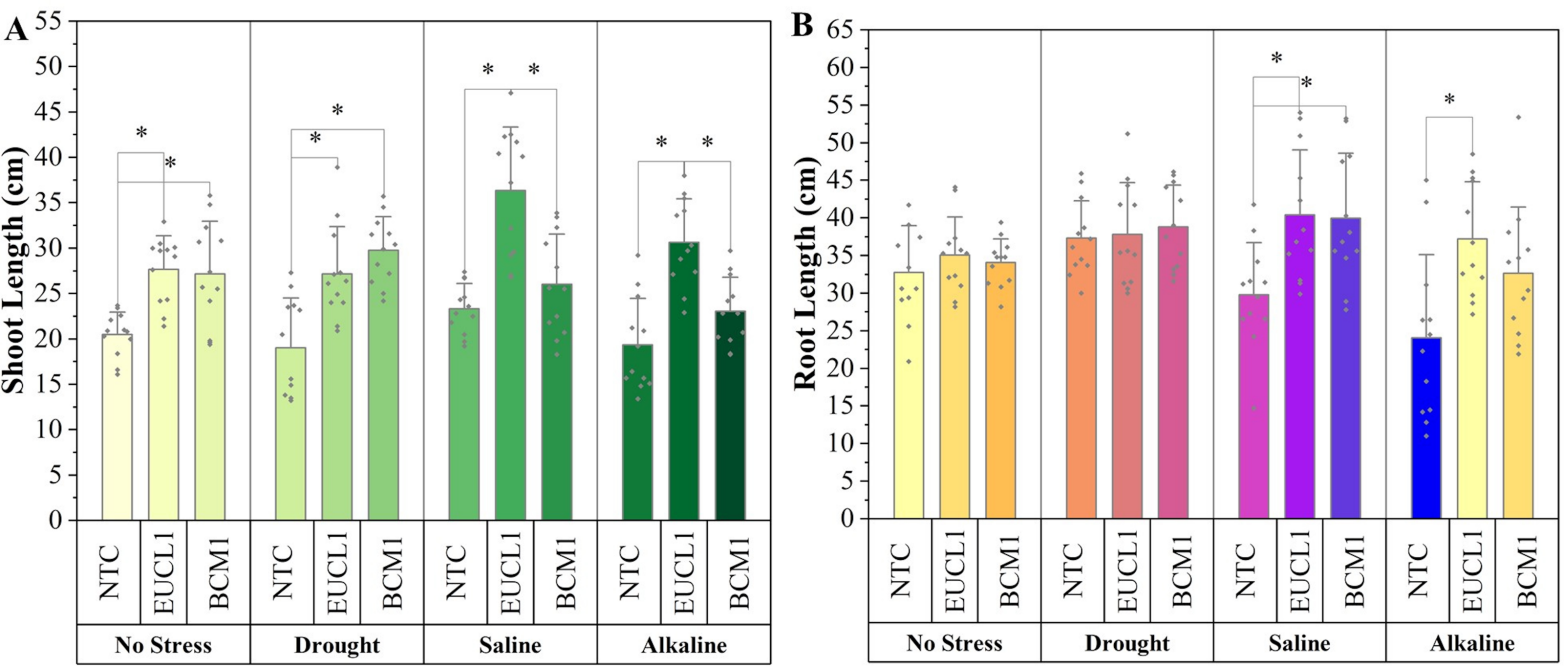
Enhanced maize lengths with *Cladophialophora inabaensis* EUCL1 and *Exophiala* sp. BCM1 treatments under all tested conditions. (A) Shoot length; (B) root length. Bars represent the mean values, with whiskers indicating the standard deviation (SD). Diamonds depict the data distribution, and asterisks (*) above connecting lines indicate significant differences between the two connected treatments at the 0.05 significance level.

Figure 5A shows the shoot length of maize cultivated under various soil conditions. Under no-stress and drought conditions, *C. inabaensis* EUCL1 and *Exophiala* sp. BCM1 significantly outperformed NTC. *Cladophialophora inabaensis* EUCL1 showed the highest increase under no-stress conditions, increasing by 34.94% compared with NTC. Under drought conditions, *Exophiala* sp. BCM1 increased shoot length by 56.41% compared with NTC. Under saline and alkaline salt conditions, *C. inabaensis* EUCL1 exhibited significant increases in shoot length by 55.77 and 58.32%, respectively, compared with NTC. Figure 5B shows that DSE treatments improved maize root length, although not all increases were significant. Under no-stress and drought conditions, no significant differences were observed among treatments. However, under saline and alkaline salt conditions, *C. inabaensis* EUCL1 significantly increased maize root length by 35.63 and 54.57%, respectively, compared with NTC.

**Figure 6:**
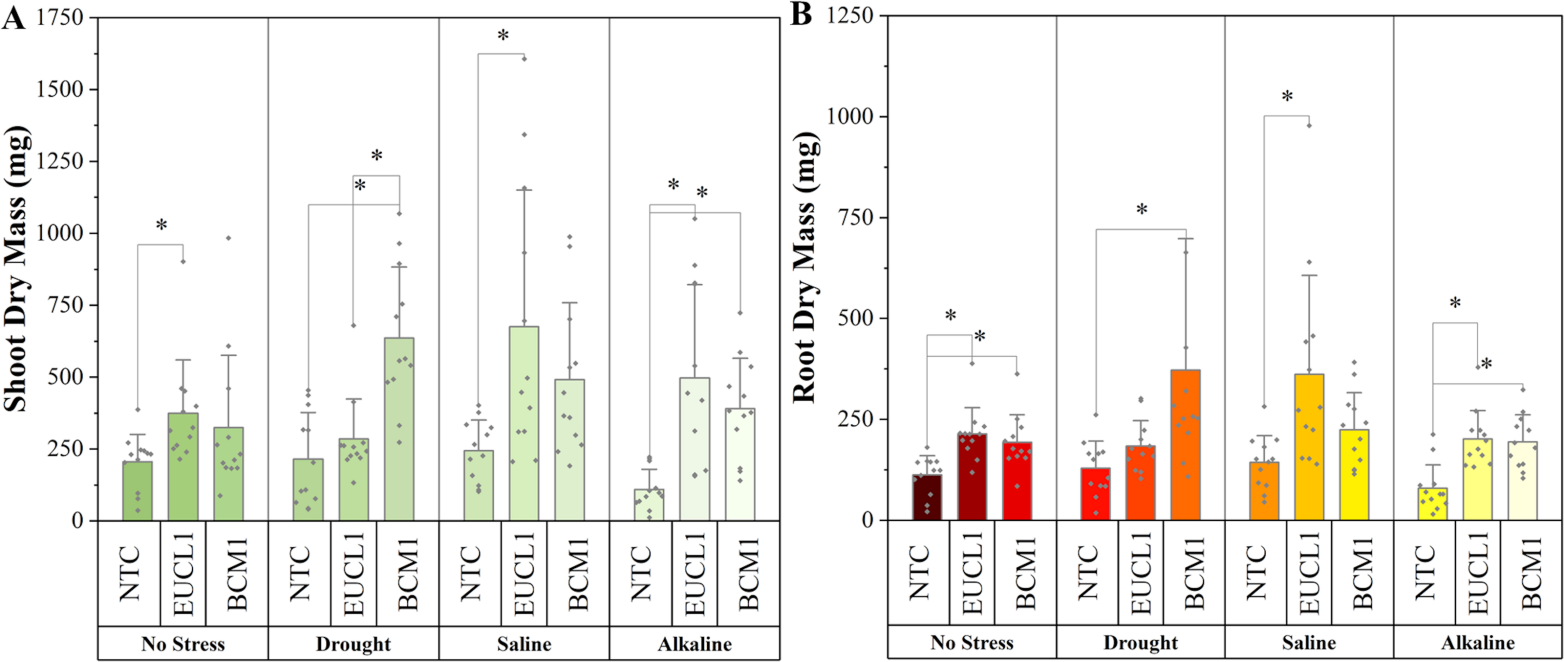
Enhanced maize dry masses with *Cladophialophora inabaensis* EUCL1 and *Exophiala* sp. BCM1 treatments under all tested conditions. (A) Shoot dry mass; (B) root dry mass. Bars represent the mean values, with whiskers indicating the standard deviation (SD). Diamonds depict the data distribution; asterisks (*) above connecting lines indicate significant differences between the two connected treatments at the 0.05 significance level.

Figure 6A demonstrates an increasing trend in maize shoot dry mass under DSE treatments. Under no-stress conditions, *C. inabaensis* EUCL1 significantly increased maize shoot dry mass compared with NTC. Under drought conditions, *Exophiala* sp. BCM1 showed the highest value, with a significant 196.55% increase compared with NTC. Under saline conditions, *C. inabaensis* EUCL1 showed the highest value with a significant 176.15% increase compared with NTC. Under alkaline salt conditions, both *C. inabaensis* EUCL1 and *Exophiala* sp. BCM1 significantly outperformed NTC, with *C. inabaensis* EUCL1 showing the highest increase of 352.28%. Figure 6B shows a similar trend, with DSE treatments increasing maize root dry mass, although not all increases were significant. Under no-stress *C. inabaensis* EUCL1 showed the highest value, with a 90.32% increase in root dry mass compared with NTC. Under drought conditions, *Exophiala* sp. BCM1 showed a significant increase of 188.21% compared with NTC. Under saline conditions, *C. inabaensis* EUCL1 showed the highest value with a significant 152.77% increase compared with NTC. Under alkaline salt conditions, both DSE treatments significantly increased root dry mass compared with NTC, with *C. inabaensis* EUCL1 showing the greatest increase of 153.3%.

**Figure 7:**
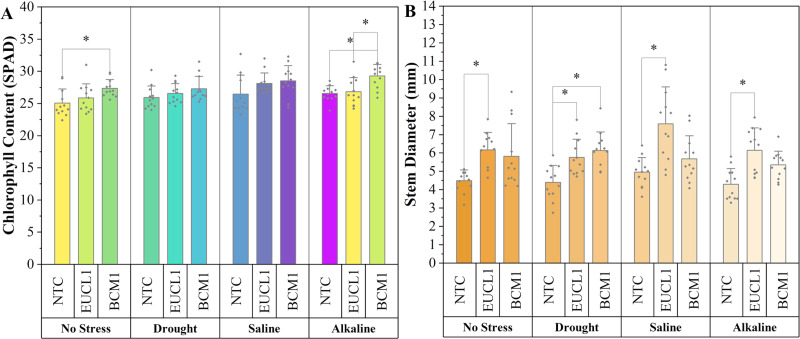
Enhanced maize chlorophyll content and stem diameter with *Cladophialophora inabaensis* EUCL1 and *Exophiala* sp. BCM1 treatments under all tested conditions. (A) Chlorophyll content; (B) stem diameter. Bars represent the mean values, with whiskers indicating the standard deviation (SD). Diamonds depict the data distribution; asterisks (*) above connecting lines indicate significant differences between the two connected treatments at the 0.05 significance level.

Figure 7A shows the chlorophyll content of maize in various environments, with all DSE treatments leading to higher values than NTC. Under no-stress conditions, *Exophiala* sp. BCM1 significantly increased the chlorophyll content by 9.1% compared with NTC. Under alkaline salt conditions, *Exophiala* sp. BCM1 also increased the chlorophyll content by 10.23% compared with NTC. However, no significant differences were observed under drought or saline conditions. Figure 7B illustrates the maize stem diameter, which increased with DSE treatments. *Cladophialophora inabaensis* EUCL1-treated plants had the largest stem diameter across no-stress, saline, and alkaline salt conditions, with increases of 37.42, 53.33, and 43.36%, respectively, compared with NTC. Under drought conditions, *Exophiala* sp. BCM1 showed the highest value, with a significant increase of 39.86% compared with NTC.

**Figure 8:**
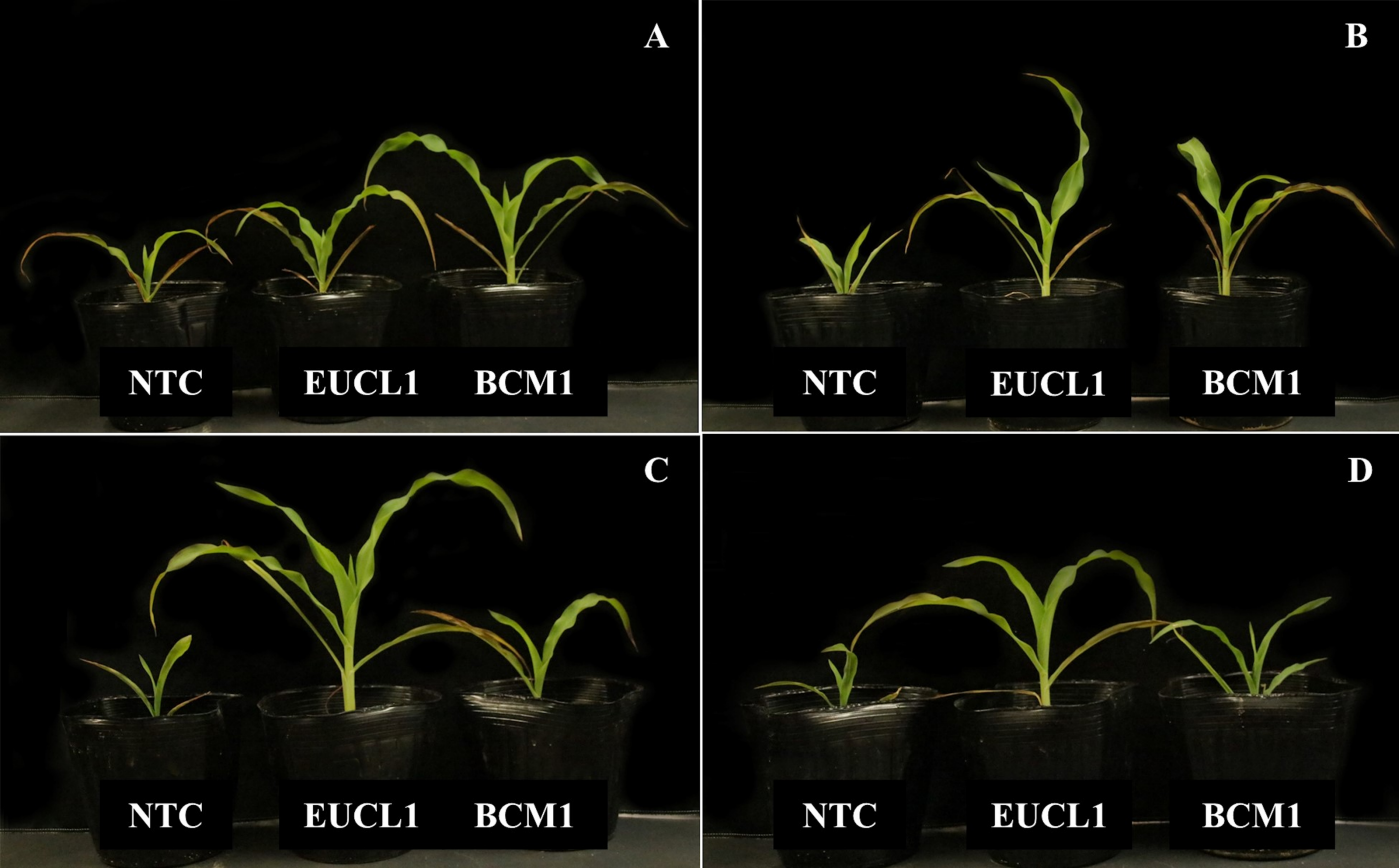
Differences in maize growth performance under various conditions, with plants treated with *Cladophialophora inabaensis* EUCL1 and *Exophiala* sp. BCM1 exhibiting improved overall growth. (A) No-stress; (B) drought stress; (C) saline stress; (D) alkaline salt stress.

As corroborated by Figs. 8A–8D, the incorporation of DSE fungi positively affects the growth of maize under various conditions, including no-stress, drought, saline, and alkaline salt environments. These figures clearly show that DSE treatments enhance maize growth compared with the NTC group.

## Discussion

This study highlights the largely unexplored potential of *C. inabaensis* EUCL1 and *Exophiala* sp. BCM1 to enhance maize growth under drought, saline, and alkaline salt stress conditions, addressing a significant gap in the current literature on maize-DSE interactions under abiotic stress. Exposure to 10% PEG, 100 mM NaCl, and 8 mM Na_2_CO_3_ resulted in marked reductions in length and dry biomass of the maize variety used, confirming its susceptibility to drought, neutral saline, and alkaline salt stress. In an *in vitro* setting, *C. inabaensis* EUCL1 significantly enhanced maize growth under no-stress and drought conditions, indicating its potential role in improving drought tolerance. Under saline and alkaline salt stress conditions, both isolates showed significant growth-promoting effects, especially in enhancing root development. Soil-based examination supported the beneficial effects of *C. inabaensis* EUCL1 and *Exophiala* sp. BCM1 in more realistic environmental settings. Both isolates enhanced germination rates compared with NTC, suggesting that their positive effects are notable as early as the germination stage. *Cladophialophora inabaensis* EUCL1 consistently enhances maize morphological traits under no-stress, saline, and alkaline salt conditions. *Exophiala* sp. BCM1 shows stronger effects in drought and offers partial support under alkaline salt stress. The chlorophyll content was improved under *Exophiala* sp. BCM1 treatment, alleviating one of the common adverse effects associated with climate change-induced stressors.

In line with our results, *Exophiala pisciphila* H93 was previously shown to promote maize biomass accumulation, improve photosynthetic performance, and alleviate the adverse effects of heavy metal stress (Xu et al., 2020). The general increase in dry mass observed in DSE-inoculated maize under stress conditions indicates a successful plant-fungal interaction, likely contributing to the mitigation of abiotic stress effects. Given that organic soil was used, the enhanced maize overall growth might be attributed to the ability of DSE fungi to promote nutrient acquisition and increase nutrient bioavailability by degrading complex organic compounds into more accessible forms (Mandyam & Jumpponen, 2005; Newsham, 2011; Surono & Narisawa, 2017). Inoculation with DSE fungi was reported to enhance host-plant tolerance to drought stress by altering the root morphology, increasing root length, and promoting root biomass accumulation (He et al., 2021; Ye et al., 2024). Similarly, the current study observed significant increases in root length and dry mass in DSE-inoculated maize under drought conditions, further suggesting improved water and nutrient uptake that may contribute to enhanced aboveground performance.

Beyond drought stress, the positive effects of *C. inabaensis* EUCL1 and *Exophiala* sp. BCM1 inoculation extended to saline stress. These results further contribute to the limited understanding of the role of DSE fungi to support maize growth under saline stress. Previous research demonstrated that inoculation with endophytic fungi promotes root development, assisting in effective nutrient acquisition and enhanced saline stress tolerance by reducing oxidative damage and maintaining ionic and metabolic balance (Ali et al., 2022; Siddiqui et al., 2022). The current study revealed a significant increase in root length and dry mass of maize inoculated with *C. inabaensis* EUCL1 and *Exophiala* sp. BCM1, suggesting enhanced tolerance to saline stress while mitigating the inhibitory effects of salt accumulation on cell division and elongation (Rahnama et al., 2011). Improvement in root traits reflects the ability of DSE fungi to modify root architecture, and thereby enhance nutrient uptake, facilitate water absorption, and thus contribute to increased aboveground biomass accumulation (Gupta et al., 2020).

While neutral salts primarily disrupt ionic balance and osmotic potential, alkaline salt stress presents additional challenges due to elevated pH levels, which reduce nutrient ion availability and directly damage root cell membranes, impairing their structural and functional integrity. Alkaline salts such as NaHCO_3_ and Na_2_CO_3_ impose dual stresses of high pH and salinity, causing more severe damage to plants than neutral salts like NaCl (Shi & Sheng, 2005). This study is the first to investigate the use of DSE fungi to alleviate alkaline salt stress in maize, offering an environmentally friendly solution to a critical but underexplored area of research. The findings demonstrate that alkaline salt stress significantly reduced maize overall growth, being consistent with previous reports showing that alkaline conditions have more detrimental effects on photosynthesis and growth than neutral salinity (Guo et al., 2017). These adverse effects are likely linked to Na^+^ accumulation, structural damage of chloroplast, and impaired chloroplast function (Yang et al., 2008; Yang et al., 2009). Notably, inoculation with *Exophiala* sp. BCM1 significantly enhanced the chlorophyll content under alkaline salt conditions, while both DSE fungi improved overall maize growth compared with uninoculated control. Available strategies to mitigate alkaline salt stress such as the application of jasmonic acid, silicon, or genetic modification, often involve complex or resource-intensive implementation (Abdel Latef & Tran, 2016; Mir et al., 2018; Fatima et al., 2021). In contrast, this study highlights DSE inoculation as a sustainable and practical strategy to mitigate alkaline salt stress in maize.

The current study represents the first report demonstrating the potential of *C. inabaensis* EUCL1 and *Exophiala* sp. BCM1 to mitigate drought, saline, and alkaline salt stress in maize. The novelty lies in revealing the ability of DSE fungi to support overall maize growth under both neutral saline and alkaline salt conditions. The consistent positive effects observed across both *in vitro* and soil-based cultivation systems underscore their potential use as biological agents to enhance the resilience of maize to climate-related stressors. While this study offers valuable insights into the potential of these two DSE isolates to improve maize growth under abiotic stress, it is limited to dry biomass, length, stem diameter, and chlorophyll content observations. Further research is needed to investigate the physiological, biochemical, and molecular mechanisms underlying their beneficial effects. In addition, exploring the effects of co-inoculation and performance of these fungi under combined stress conditions will be essential to maximize their application potential. Despite these limitations, our results contribute significantly to the growing body of knowledge on the role of DSE fungi in sustainable agriculture. Especially, DSE inoculation offers a promising low-input strategy to support maize cultivation in climate-change-affected agricultural land.

## Conclusion

This study highlights the beneficial roles of DSE *Cladophialophora inabaensis* EUCL1 and *Exophiala* sp. BCM1 to enhance maize growth under drought, saline, and alkaline salt stress. Inoculated plants exhibited increased root and shoot length, biomass, stem diameter and chlorophyll content, suggesting improved stress tolerance mediated through successful plant-fungal interactions. This study is the first to report the beneficial effects of DSE fungi in enhancing maize tolerance to drought, salinity, and alkalinity, addressing a critical gap in the existing literature. These findings support the potential use of DSE as sustainable, low-input strategy to enhance the resilience of maize under climate-related stresses.

## Additional Information and Declarations

### Data Availability

The following information was supplied regarding data availability: Raw data is available at Figshare: Innosensia, Ni Luh Putu Citra; Lu, Haoyue; Narisawa, Kazuhiko (2025). Supplementary_Raw Data. figshare. Dataset. https://doi.org/10.6084/m9.figshare.28190411.v1
